# Supplementary material for: HIF-Dependent CKB Expression Promotes Breast Cancer Metastasis, Whereas Cyclocreatine Therapy Impairs Cellular Invasion and Improves Chemotherapy Efficacy
Source: Cancers (Basel). 2021 Dec 22;14(1):27. doi: 10.3390/cancers14010027 (PMC8749968; doi:10.3390/cancers14010027)
Supplement: Supplementary file 1 [file cancers-14-00027-s001.zip › cancers-1496148-supplementary.pdf]

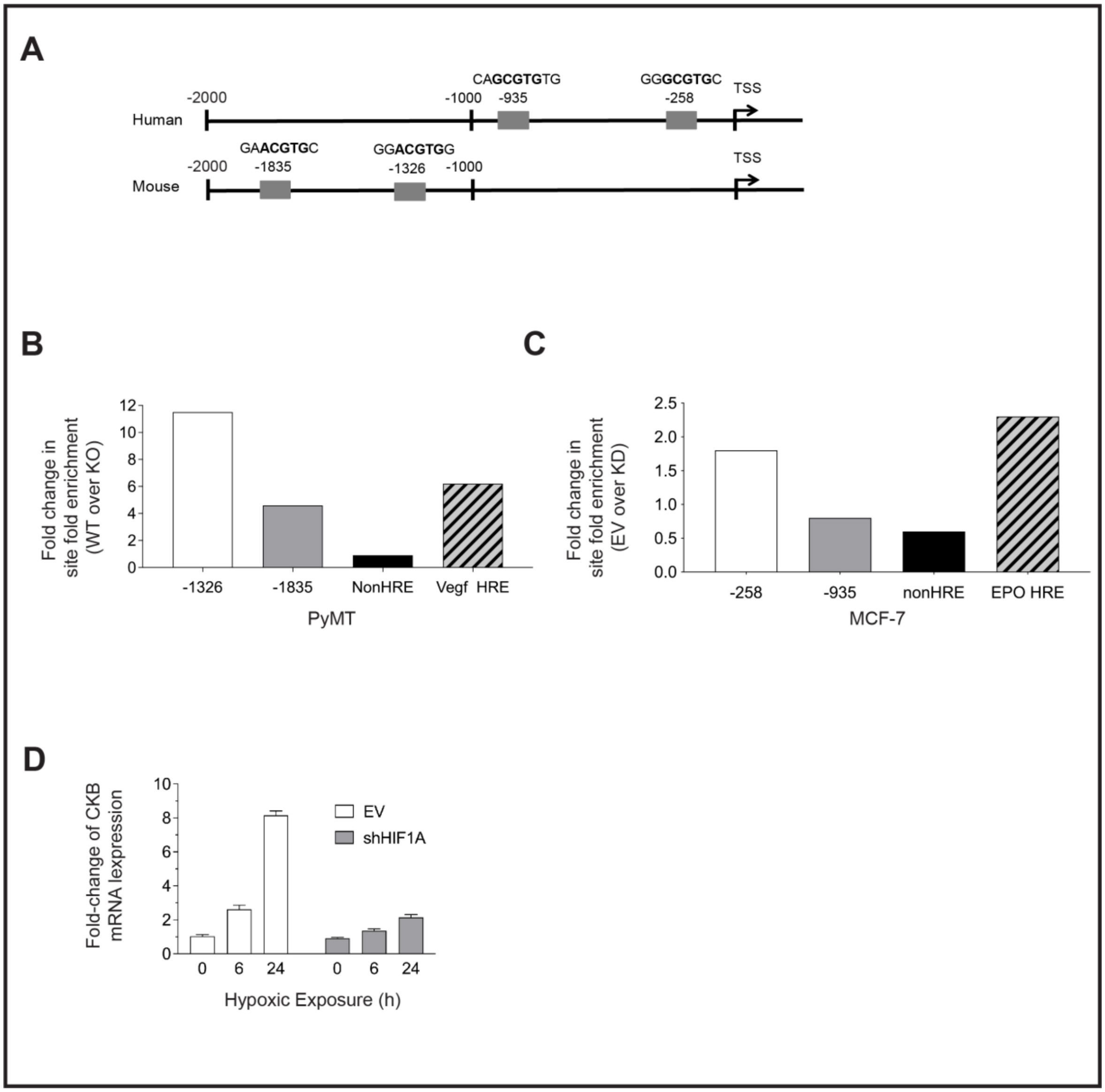

Supplementary Figure S1. Chromatin immunoprecipitation (ChIP) analysis of the *Ckb*/*CKB* proximal promoter in PyMT and MCF-7 breast cancer cells. **A**. A schematic representation of two putative hypoxia response elements (HREs) identified by the JASPAR database in either the mouse (*Ckb*) or the human (*CKB*) gene proximal promoter. **B-C**. All cells were cultured to ~80% confluence and then exposed to hypoxia for 24 h (0.5% O_2) prior to the isolation of chromatin. ChIP assays were performed with antibodies to HIF-1α antibodies (cat# H1alpha67, ab1, AbCAM, Cambridge, UK) and qPCR was conducted on the isolated DNA to determine HIF1α recruitment. Binding enrichment represented at each HRE site is expressed as the fold change between HIF-1 wild type PyMT cells (**B**), or MCF-7 empty vector (**C**) cells relative to either HIF-1 KO PyMT cells (**B**) or MCF-7 shHIF1A knockdown (KD) cells (**C**), respectively, after normalizing to the IgG control. As internal assay controls for the PyMT cells, ChIP was also performed for a known HRE in the mouse *Vegf* promoter [35] or using an intron within Ckb with no identified HRE sites (nonHRE). As internal assay controls for MCF-7 cells, ChIP for HIF-1α was performed in a known HRE site in the EPO 3′ UTR [36], or using an intron within *CKB* with no identified HRE sites. D. *CKB* mRNA levels were measured by qPCR in MCF-7 EV and shHIF1A cells cultured for 0, 6 or 24 h of hypoxia. The fold change in *CKB* mRNA expression was calculated after normalization for loading to *PPIA*.

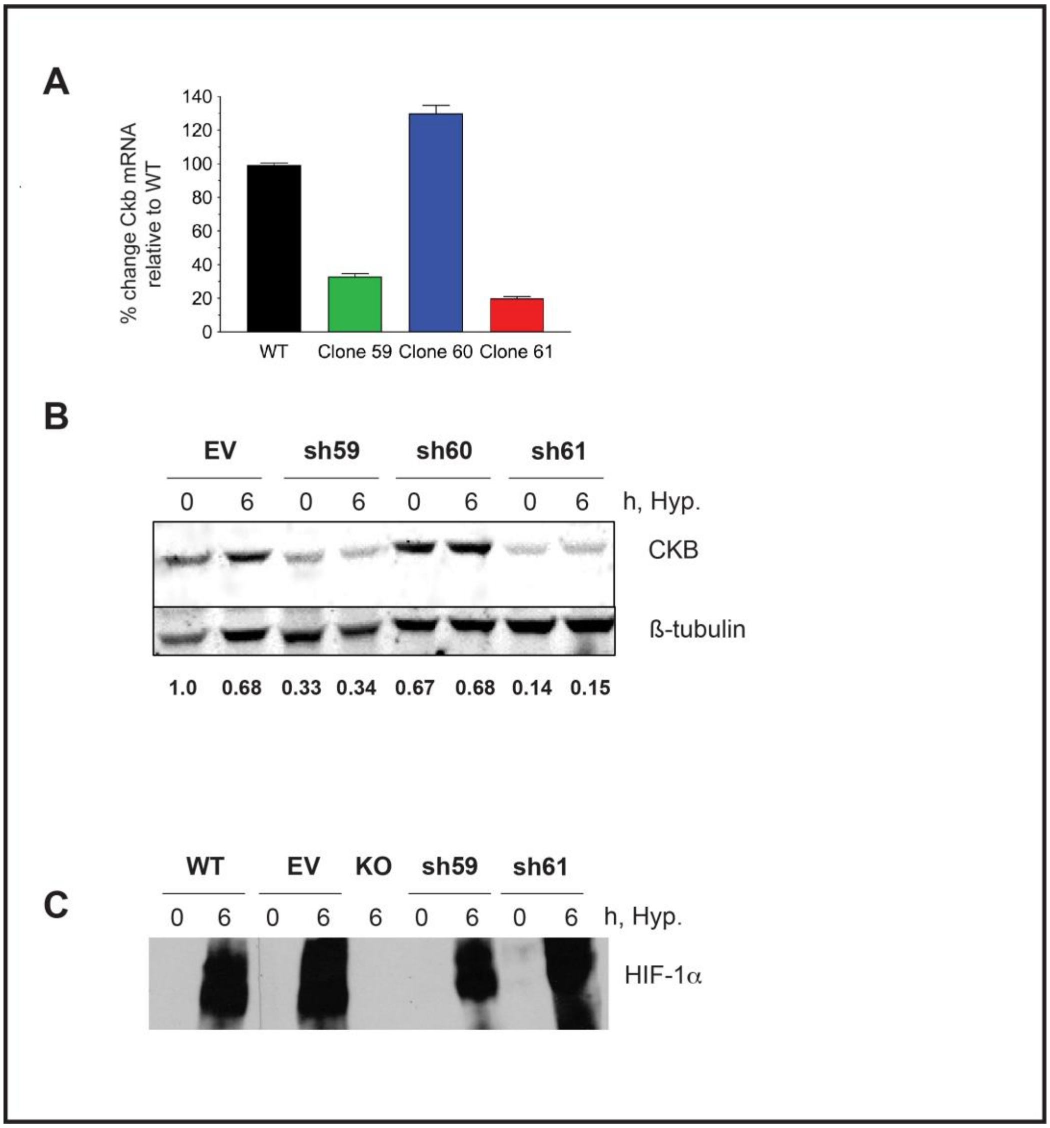

Supplementary Figure S2. Comparing gene knockdown efficiency of *Ckb* shRNA constructs in PyMT cells. A. HIF-1 WT PyMT cells were transfected with three different pLKO.1-puro-shRNA targeting viral backbones to screen for deletion efficiency at the mRNA level by qPCR (n = 4 technical replicates/cell line) when cells were cultured at normoxia. **B**. Western blot analysis of CKB protein levels using replicate plates from (**A**), along with EV PyMT cells (expressing empty pLKO.1-puro); β-tubulin is used as a loading control. The EV at 0 h hypoxia sample was set to 1.0 and ImageJ was used to evaluate densitometry values of CKB protein. **C**. Western blotting of HIF-1α in WT parental, HIF-1 WT + EV (EV), HIF-1 KO parental, or *Ckb* KD sh59 and sh61 cells cultured at normoxia (excluding HIF-1 KO) or for 6 h at hypoxia following transduction with pLKO.1-puro-based recombinant lentiviruses, and the selection of stable shRNA KD pools. HIF-1α is present as a ubiquitinylated protein smear after exposure to hypoxia.

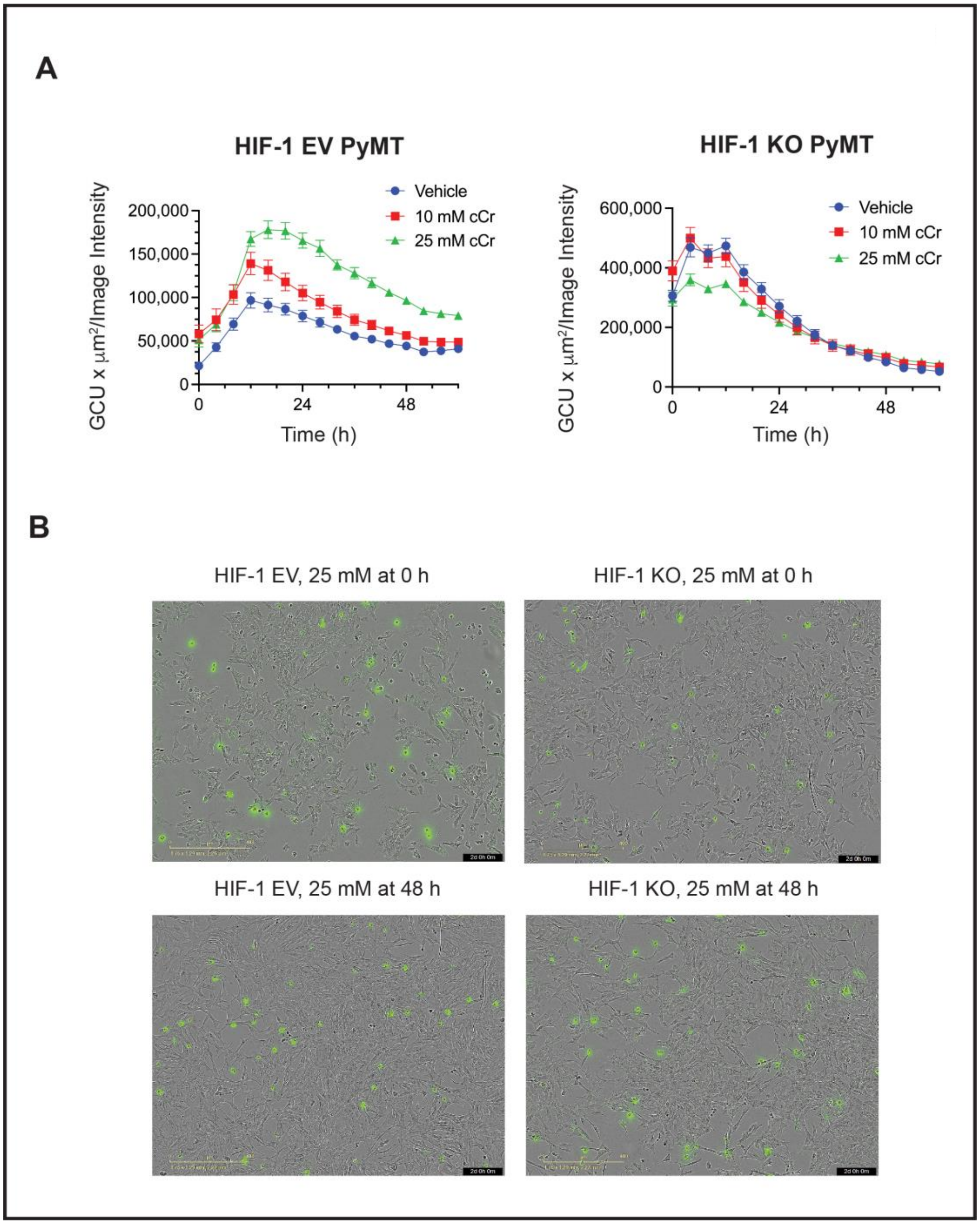

Supplementary Figure S3. CytoTox Green assays to measure cell viability after exposure to 15 mM or 25 mM cCr for up to 48 h. A. PyMT EV cells used in wound healing, invasion, and cell cycle progression experiments presented in Figure 4 were grown in 2% FBS (standard growth medium) and then exposed to either 15 mM cCr (the ~IC_{50}) or 25 mM cCr in the presence of CytoTox Green reagent. The intensity of CytoTox green dye incorporated by dead cells was enumerated over time using the IncuCyte S3 live-cell imager. **B**. Although HIF-1 EV cells appeared to be more sensitive to cCr treatment (**A**), they also had a higher basal level of cell death on a per cell basis than HIF-1 KO cells at t = 0 h. Example well scan images for each genotype are shown at t = 0 h (1 h after addition of CytoTox and when scanning began) and at t = 48 h (experimental endpoint). Overall, the per cell level of CytoTox Green is approximately equivalent between EV and HIF-1 KO cells in the presence of 25 mM cCr.

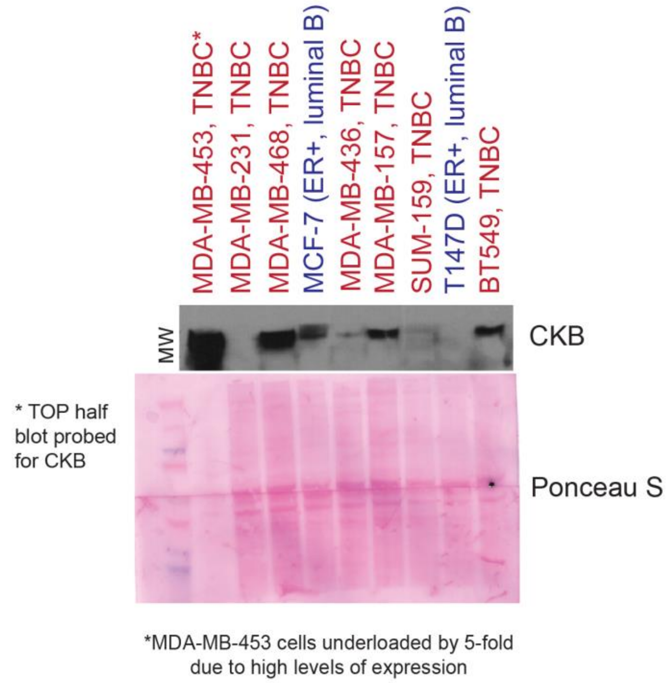

Supplementary Figure S4. Comparison of CKB levels in additional human breast cancer cell lines. A. Western blot comparing CKB expression between TNBC cells (red font: MDA-MB-453, MDA-MB-231, MDA-MB-468, MDA-MB-436, MDA-MB-157, SUM-59, BT-549) and two ER-positive luminal B models (blue font), MCF-7 cells, known to express CKB, and T47D cells. Of note, the MDA-MB-453 sample was underloaded by ~5-fold due to high levels of expression (refer to Figure 7A); for the other samples, loading is equivalent as shown by Ponceau S staining of the whole blot; however, only the top half of the blot was probed for CKB.

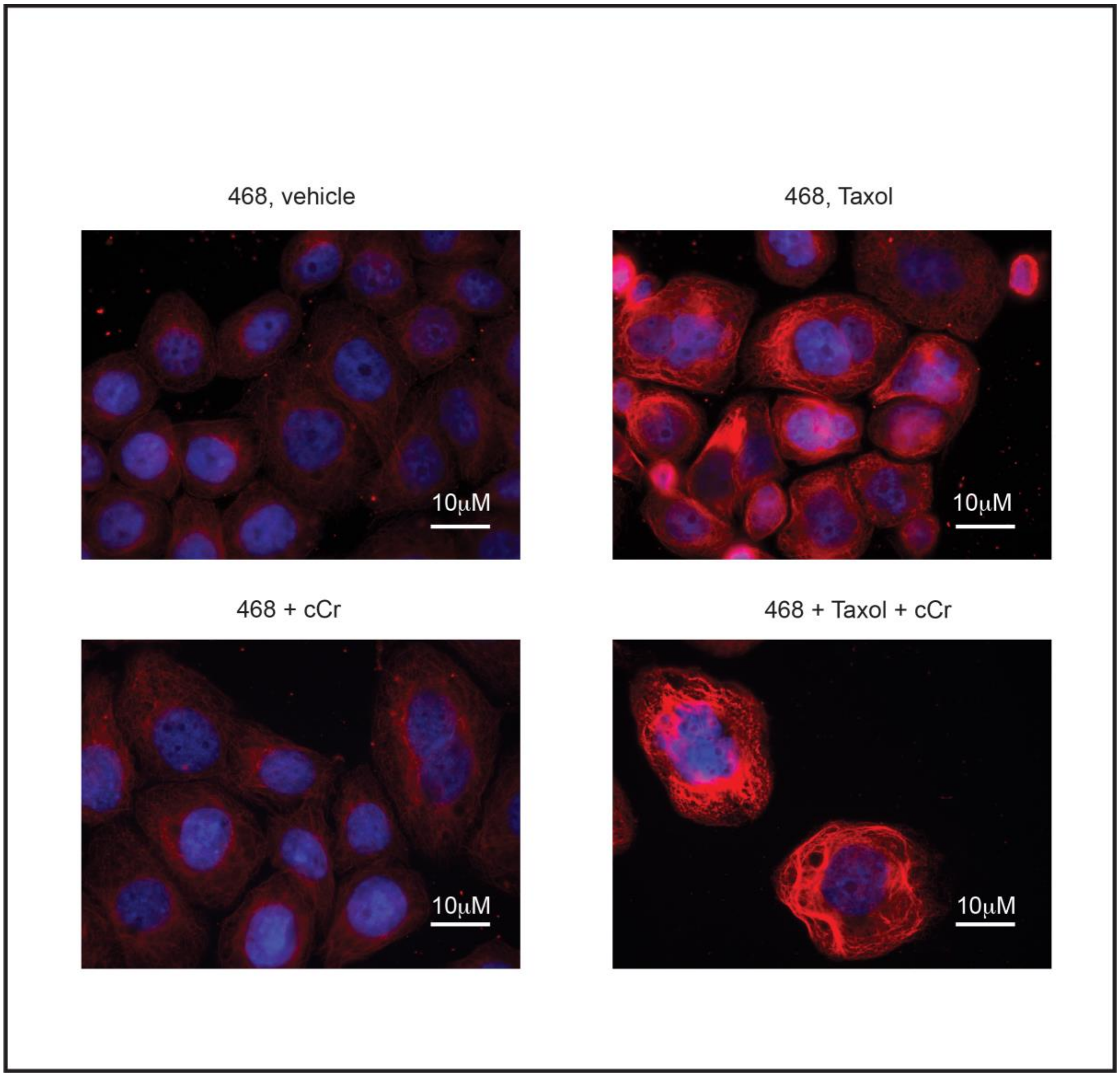

Supplementary Figure S5. Immunostaining of invadopodia coverslips at the experimental endpoint. A. Example images of coverslips used for invadopodia assays for the BT549 TNBC cell line. During invadopodia formation, BT549 cells were exposed to either vehicle (left panel) or to 25 mM cCr (~IC_{50}; right panel) for 48-50 h (green=gelatin; red=cortactin; blue=DAPI). Scale bar represents 20 μM. B. MDA-MB-231 cells ectopically expressing mCKB readily formed invadopodia (Figure 7). At the study endpoint, coverslips containing invadopodia were immunostained with CKB antibodies. The image represents the same area imaged with either a short or a long exposure time in the red channel with the green laser intensity also minimized. As shown with the longer exposure image, mCKB can be found along the periphery of the cell and in the cell projections overlapping with invadopodia, as well as in the cytoplasm and in the nucleus; the scale bar represents 20 μM.

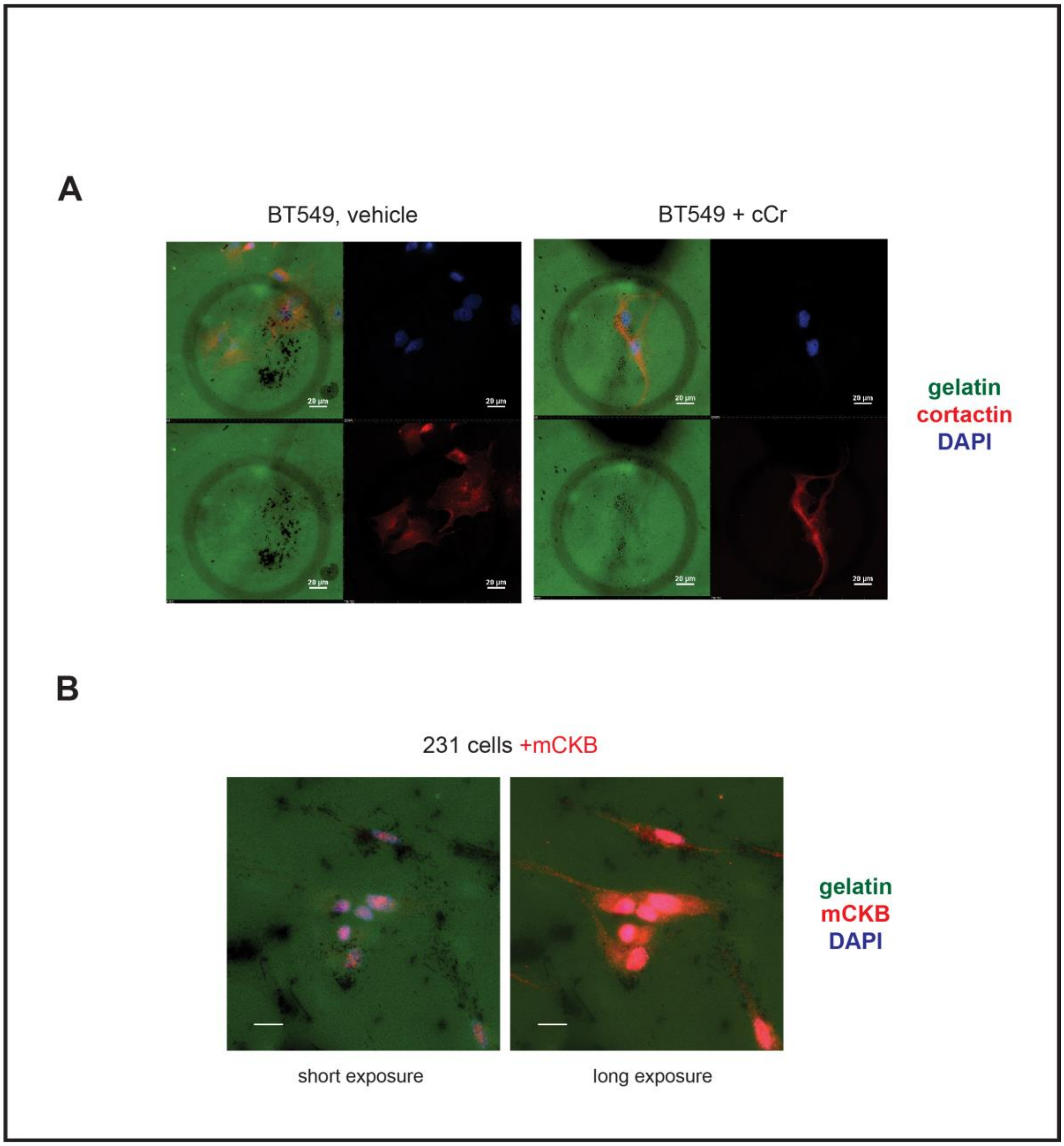

Supplementary Figure S6. Tubulin networks following Taxol treatment in combination with cCr. MDA-MB-468 cells were plated into ibidi multi-chamber well polymer slides, allowed to adhere for 48h, treated with either 20 mM cCr, 10 nM Taxol, or both drugs for 48 h, then washed, fixed, and immunostained with alpha-tubulin (red) and counterstained with DAPI. All images were captured on a Nikon ECLIPSE Ti2 microscope at the same laser intensities and exposure times at 400 x magnification; scale bars represent 20 µM.

Supplementary Tables

Table S4. Primers and Roche Universal Probe Library (UPL) FAM-labeled probes utilized in real-time PCR assays. All assays were designed using the Roche Universal ProbeLibrary Assay Design Center.

Gene	Forward Primer	Reverse Primer	UPL ID
Ckb (murine)	gcaagcacaggcatccat	cgcagcttctgcgtattatg	70
CKB (human)	cctgcccagaaatgaagc	gcactgcccaggcaataa	38
Ints3 (murine)	gtggctgttattgactctgcac	caggttccccatcatcacat	17
Krt18 (murine K18)	agatgacaccaacatcacaagg	cttccagaccttggacttcct	78
PPIA (Cyclophilin A)	atgctggacccaacacaaat	tctttcactttgccaaacacc	48

Table S5. Primary antibody source and dilution factors utilized in western blotting, immunofluorescence (IF) staining of cultured cells and DAB-based immunohistochemistry (IHC) staining of tumor tissues.

Antibody	Source and Catalog #	Dilution	Purpose
anti-CKB	AbCAM (#ab88746)	1:5,000	western
anti-β-tubulin	AbCAM (#(#ab6046)	1:10,000	western
anti-HIF-1α	Novus Biologicals (#NB100-479)	1:5,000	western
anti-PARP	Cell Signaling Technology (#9542)	1:5,000	western
anti-CKB	ThermoFisher (PA5-21382) or Santa Cruz, sc-271531)	1:500	IF, cells
anti-alpha tubulin	ThermoFisher (#62204)	1:250	IF, cells
anti-cortactin	Millipore (#05-0180)	1:700	IF, cells
anti-mouse AlexaFluor-488	Life Technologies (#A21202)	1:400	IF, cells
anti-rabbit AlexaFluor-594	Life Technologies (#A21203)	1:400	IF, cells
anti-rabbit-IgG-Biotin-X	Life Technologies (#A16027)	1:400	IF, cells
Strepavidin-AlexaFlour-594	Life Technologies (#S11227)	1:400	IF, cells
anti-Ki67	AbCAM (#ab-15580)	1:750	IHC
anti-CD31	Cell Signaling Technology (#77699)	1:100	IHC
anti-activated caspase3	Cell Signaling Technology (#9661)	1:100	IHC

Table S6. Primers used in chromatin immunoprecipitation (ChIP) assays in murine PyMT tumor cells and in human MCF-7 cells.

Genomic Region	IP Antibody	Forward Primer	Reverse Primer
-258 *CKB* *(human)*	HIF-1α	gatgaacccaagcgtcctc	agacctcgaggccgaaac
-935 *CKB (human)*	HIF-1α	attgctgggtcacggagtt	tacccccaacactcccagat
-1869 *CKB* non-HRE *(human)*	HIF-1α	ttcaggttttgtggggtagc	ctgttccaagccagcatttt
-1326 *Ckb (murine)*	HIF-1α	cagggctgttctggactctc	cctcacaagtgctgggatta
-1835 *Ckb (murine)*	HIF-1α	tgcctcctgtgcactttttt	agcctagcttcagagaactaatgg
-510 *Ckb* non-HRE (murine)	HIF-1α	cttggcggtgttccttagag	caggccatatcctcaagagc
EPO (human)	HIF-1α	gctggcctctggctctcatgg	cagggttggcagctgccttactg
Vegf (murine)	HIF-1α	ctggcttcagttccctggcaacatctct	cctggggtgaatgggatcctctgg

Table S7. Murine *Ckb* shRNA targeting sequences obtained from The RNAi Consortium (TRC) library purchased through Open Biosystems (Huntsville, AL, USA). The shRNAs were named after the last two digits of the Clone ID (for example, shRNA 59, 60 or 61).

Clone ID	Clone Name	Target Sequence
TRCN00000246**59**	NM_021273.2-300s1c1	cgacgtattcaaggacctctt
TRCN00000246**60**	NM_021273.2-288s1c1	cgaggagagttacgacgtatt
TRCN00000246**61**	NM_021273.2-708s1c1	gcacaatgacaataagacttt
